# Sex-specific plasma lipid profiles of ME/CFS patients and their association with pain, fatigue, and cognitive symptoms

**DOI:** 10.1186/s12967-021-03035-6

**Published:** 2021-08-28

**Authors:** Aurore Nkiliza, Megan Parks, Adam Cseresznye, Sarah Oberlin, James E. Evans, Teresa Darcey, Kristina Aenlle, Daniel Niedospial, Michael Mullan, Fiona Crawford, Nancy Klimas, Laila Abdullah

**Affiliations:** 1 Roskamp Institute, 2040 Whitfield Ave, Sarasota, FL 34243 USA; 2 James A. Haley Veterans’ Hospital, 2040 Whitfield Ave, Tampa, FL USA; 3 Institute for NeuroImmune Medicine, VAMC, GRECC, Nova Southeastern University, Miami, USA

**Keywords:** Myalgic encephalomyelitis/chronic fatigue syndrome, Lipidomic, Inflammation, Immunity

## Abstract

**Background:**

Myalgic encephalomyelitis/chronic fatigue syndrome (ME/CFS) is a complex illness which disproportionally affects females. This illness is associated with immune and metabolic perturbations that may be influenced by lipid metabolism. We therefore hypothesized that plasma lipids from ME/CFS patients will provide a unique biomarker signature of disturbances in immune, inflammation and metabolic processes associated with ME/CFS.

**Methods:**

Lipidomic analyses were performed on plasma from a cohort of 50 ME/CFS patients and 50 controls (50% males and similar age and ethnicity per group). Analyses were conducted with nano-flow liquid chromatography (nLC) and high-performance liquid chromatography (HPLC) systems coupled with a high mass accuracy ORBITRAP mass spectrometer, allowing detection of plasma lipid concentration ranges over three orders of magnitude. We examined plasma phospholipids (PL), neutral lipids (NL) and bioactive lipids in ME/CFS patients and controls and examined the influence of sex on the relationship between lipids and ME/CFS diagnosis.

**Results:**

Among females, levels of total phosphatidylethanolamine (PE), omega-6 arachidonic acid-containing PE, and total hexosylceramides (HexCer) were significantly decreased in ME/CFS compared to controls. In males, levels of total HexCer, monounsaturated PE, phosphatidylinositol (PI), and saturated triglycerides (TG) were increased in ME/CFS patients compared to controls. Additionally, omega-6 linoleic acid-derived oxylipins were significantly increased in male ME/CFS patients *versus* male controls. Principal component analysis (PCA) identified three major components containing mostly PC and a few PE, PI and SM species—all of which were negatively associated with headache and fatigue severity, irrespective of sex. Correlations of oxylipins, ethanolamides and ME/CFS symptom severity showed that lower concentrations of these lipids corresponded with an increase in the severity of headaches, fatigue and cognitive difficulties and that this association was influenced by sex.

**Conclusion:**

The observed sex-specific pattern of dysregulated PL, NL, HexCer and oxylipins in ME/CFS patients suggests a possible role of these lipids in promoting immune dysfunction and inflammation which may be among the underlying factors driving the clinical presentation of fatigue, chronic pain, and cognitive difficulties in ill patients. Further evaluation of lipid metabolism pathways is warranted to better understand ME/CFS pathogenesis.

**Supplementary Information:**

The online version contains supplementary material available at 10.1186/s12967-021-03035-6.

## Introduction

Myalgic encephalomyelitis/chronic fatigue syndrome (ME/CFS) is a multisymptom illness affecting up to 2.5 million individuals in the US population [Centers for Disease Control and Prevention (CDC), 2018]. Patients with ME/CFS experience post-exertional malaise (PEM), chronic fatigue, debilitating pain and cognitive problems that interfere with activities of daily living and negatively impact educational and earning potential of patients suffering from ME/CFS. The annual cost associated with the medical care and management of ME/CFS in the US is estimated to be 9 billion dollars [1, 2]. Another 24 billion dollars was associated with ME/CFS-related loss of productivity. The total economic impact is now considered to have reached 50 billion dollars in both medical costs and lost productivity [3, 4]. The nature and the severity of ME/CFS symptoms vary among ill patients, making it difficult to characterize and diagnose this condition. Currently, there are no approved blood biomarkers of ME/CFS that can provide an indication of the underlying biological aspects associated with ME/CFS diagnosis. As such, blood biomarkers are needed to help clinicians accurately detect ME/CFS and provide appropriate therapies for symptom management.

While the etiological origins of ME/CFS remains unknown, reports of prolonged and persistent flu-like symptoms suggest a failure to restore homeostasis of the immune system after either environmental or infectious triggers [5]. These immune alterations are characterized by decreases in the natural killer (NK) cells and increases in the circulating CD4+ and CD8+ T-cells, T-regulatory cells (Tregs) and memory B-cells in subjects with ME/CFS compared to healthy individuals [6, 7]. Immune dysfunction in ME/CFS is also supported by dysregulation of pro-inflammatory and anti-inflammatory cytokines in the blood and cerebrospinal fluid (CSF) of ME/CFS patients [8, 9]. These studies support a role of altered immune and inflammatory responses in ME/CFS, which worsen with increasing chronicity of this illness [10–12].

Immune cells, including T-cells, NK and macrophages, use lipids for supporting energy demands as well as for generating lipid mediators for activating and regulating inflammatory responses. For instance, the omega-6 arachidonic acid (AA) undergoes enzymatic oxidation to generate eicosanoids which activate pro-inflammatory pathways. On the contrary, omega-3 docosahexaenoic acid (DHA) can be metabolized to produce resolvins which promote an anti-inflammatory response. These essential fatty acids are abundant in phospholipids (PL) and neutral lipids [13–16]. Recent metabolomics studies provide an excellent overview of disturbances in PL, cholesterol ester (CE), cholesterol and triglycerides (TG) in ME/CFS [17–20]. However, an evaluation of the omega-6 and omega-3 fatty acid content of these lipids has not been examined thoroughly and could potentially be useful in identifying lipid signatures that are specific to immune and inflammatory processes associated with ME/CFS.

A recent study showed that circulating CD4+ and CD8+ T cells from ME/CFS patients exhibit reduced mitochondrial membrane potential and metabolic remodeling capacity, suggestive of immune system exhaustion [21]. Additional support for mitochondrial bioenergetics deficits in ME/CFS comes from studies showing alterations in pathways related to metabolism of energy substrates, amino acid, nucleotide, choline, carnitine, fatty acid and complex lipids, such as PL, sphingolipids and neutral lipids [17–20]. As such, evaluation of lipids that are specific to mitochondria function such as acylcarnitines (CAR) may be helpful for understanding the underlying metabolic changes associated with ME/CFS.

Given the importance of lipids and lipid metabolites in immunity and inflammatory pathways as well as in bioenergetics, we hypothesized that lipid profiles associated with these functions will differ between ME/CFS patients and controls. Hence, we quantified these lipids in plasma from ME/CFS patients and controls with similar age, sex and general demographics using high resolution high-performance liquid chromatography-mass spectrometry (HPLC/MS) lipidomic technologies. This study provides an indication of an association between plasma lipid differences between ME/CFS and controls. Furthermore, lipid biomarker signatures associated with ME/CFS may be helpful to clinicians in determining a suitable course of action for managing and treating ME/CFS.

## Methods

### Cohort description

Using a cross-sectional design, plasma samples were collected from 50 controls and 50 ME/CFS patients. To examine sex differences, approximately 50% of our cohort were females and 50% were males (Table 1). Research approvals were obtained from the Institutional Review Board (IRB 4 987.81). Trained and certified staff conducted the informed consent process using the International Committee on Harmonization of Good Clinical Practice (ICH-GCP) guidelines. Subject eligibility was determined using the inclusion criteria which stipulated that a patient must demonstrate symptoms from the following 6 categories: fatigue, post-exertional malaise (PEM) and/or fatigue, pain, neurologic/cognitive manifestations, and autonomic, neuroendocrine, or immune manifestations. Diagnosis of ME/CFS was made according to the Fukuda (1994) and Carruthers (2003) case definitions. Patients were also expected to demonstrate cognitive impairment or orthostatic intolerance. Healthy controls were self-defined as healthy, not meeting any of the case definitions of ME/CFS, sedentary and were similar to cases for age, sex, race/ethnicity and body mass index (BMI). Another eligibility requirement included being between 18 and 65 years of age. Both sexes and different ethnicities were recruited. Participants were excluded if they met the fourth edition of the Diagnostic and Statistical Manual of Mental Disorders criteria (DSM-IV criteria) for schizophrenia, bipolar disorder, or substance abuse. Participants with a history of heart disease, chronic obstructive pulmonary disease, malignancy, or other systemic disorders, hospitalized for affective disorders or diagnosed with any other illness having implied receipt of medical treatment that would explain chronic fatigue and/or modulate neuroendocrine or immune indicators (e.g., Lyme disease, renal dialysis, cancer) were excluded. For sub-analysis of lipid profiles with symptoms, variables from the DePaul Fatigue questionnaire were used to evaluate physical pain, headache, cognitive difficulties, and fatigue severity [22]. Data on DHA supplementation was collected based on self-reporting of omega-3 or fish oil use. Individuals who did not report concomitant medication use were considered as not taking DHA supplement.

**Table 1:** Descriptive table of demographic variables of ME/CFS patients and controls

Characteristics	Controls (n = 50)			ME/CFS (n = 50)			Total (n = 100)		
	Females	Males		Females	Males		Females		Males
Sex (n)	25 (50%)	25 (50%)		25 (50%)	25 (50%)		50 (50%)		50 (50%)
Age (Mean $\mp$SD)	44.7 (12.2)	34.5 (9.7)		48.4 (9.9)	38.7 (12.9)		46.6 (11.2)		36.6 (11.5)
BMI $\mp$ (Mean $\mp$ SD)	27.2 (2.8)	27.4 (3.6)		26.9 (4.9)	26.2 (4.7)		27.0 (4.0)		26.8 (4.2)
Race/ethnicity									
Caucasian (n)	17 (34%)	18 (36%)		23 (46%)	20 (40%)		40 (40%)		38 (38%)
Non-caucasian (n)	8 (16%)	7 (14%)		2 (4%)	5 (10%)		10 (20%)		12 (24%)
Symptoms									
Fatigue (n)	0	1 (2%)		21 (42%)***	23 (46%)***		21 (21%)		24 (24%)
Physical pain (n)	2 (4%)	8 (16%)		24 (48%)***	18 (36%)***		26 (26%)		26 (26%)
Headache (n)	2 (4%)	0		18 (36%)***	14 (34%)***		20 (20%)		14 (14%)
Cognitive difficulties (n)	0	1 (2%)		17 (34%)***	23 (46%)***		17 (17%)		24 (24%)
Supplement									
DHA (n)	1 (2%)	1 (2%)		5 (10%)	3 (6%)		6 (6%)		4 (4%)

Fisher exact test was used to calculate p-values

*** p-value < 0.001 for the comparison of CFS patients versus their respective controls

### Lipidomic analyses

#### LC/MS analysis of phospholipids, neutral lipids and sphingolipids

The procedure of lipid extraction was previously described [23] and lipid levels were separated using a Thermo Scientific™ EASY-nLC 1000 Liquid Chromatograph interfaced to a Thermo Scientific LTQ Orbitrap Mass Spectrometer equipped with a Nanospray Flex™ Ion Source. Samples were dissolved in 70% solvent A (27% isopropyl alcohol, 42% water, 21% acetonitrile, 0.1% formic acid, 10 mM ammonium formate), and 30% solvent B (90% isopropyl alcohol, 10% acetonitrile, 0.1% formic acid, 10 mM ammonium formate) and loaded onto an Acclaim PepMap 100, 75 μm × 2 cm nanoViper C18, 3 μm, 100 Å trapping column at 3 µL/min. The loaded samples were chromatographed on an Acclaim PepMap RSLC, 75 μm × 15 cm nanoViper C18, 2 μm, 100 Å analytical column, running the following gradient at a constant flow rate of 250nL/min. The starting conditions were 30% solvent B in solvent A, then from 1 to 50 min program linearly from 50 to 98% B, then switched to 30% B from 50 to 65 min. All samples were run in triplicate along with a blank and quality control (QC) sample. Data were acquired in full scan, both positive (mass range of m/z 130–2000) and negative (mass range of m/z 220–2000) ion modes at a resolution of 30,000. The heated capillary was maintained at 200 °C, with a spray voltage of 1500 V. A maximum injection time of 200 ms was used with 13 microscans/acquired scan.

### Acylcarnitines

Each sample (50 μL) was spiked with 5 μL of deuterium labeled carnitine internal standards (IS) (NSK-B, Cambridge Isotope Laboratories Inc) in a 1.5 mL low retention microcentrifuge tube (Eppendorf) and proteins precipitated by addition of 500 μL of 25% methanol in acetonitrile. Plasma samples were vortexed and centrifuged at 10,000×*g* at 4 °C for 20 min. Supernatants were then transferred into clean 1.5 mL microtubes (Eppendorf) and dried under a vacuum. The dried pellets were reconstituted in 100 μL of mobile phase A consisting of 90% of acetonitrile, 5% of water and 5% of 100 mM ammonium formate, briefly sonicated in a bath sonicator and centrifuged at 10,000×*g* at 4 °C for 5 min and transferred into autosampler vials. Samples were injected into a Kinetex 2.6 μm HILIC 100 Å, 100 × 2.1 mm LC column, with a flow rate of 250 µL/min, for a lipid chromatographic separation using a Shimadzu HPLC system connected to a Thermo Scientific™ Q Exactive™ hybrid quadrupole-orbitrap mass spectrometer. The solvent gradient conditions used were 20% B, then 0–5 min 22% B, 5–10 min 40%B, 10–13 min 60% B, 13–15 min 80% B, where solvent B consisted of 50% of acetonitrile, 45% of water and 5% of 100 mM ammonium formate. Finally, the column was re-equilibrated for 5 min at 20% B. All samples were run in triplicate along with a blank and a quality control (QC) sample. Data were acquired in full scan in positive ionization mode, m/z 140–600, using a parallel reaction monitoring (PRM) for acylcarnitines of interest at a resolution of 17,500. The heated capillary was maintained at 253 °C, with a spray voltage of 3500 V.

### Ethanolamides

Fifty microliters of plasma were spiked with stable isotope labeled ethanolamide species as well as 10 μL of butylated hydroxytoluene in methanol (10 mg/mL) to prevent auto-oxidation. Proteins from each sample were then precipitated in 150 μL ice-cold methanol. Samples were centrifuged at 10,800 rpm for 10 min at 4 °C and supernatants were transferred into 1.5 mL low retention microcentrifuge tubes. Supernatants were further diluted by the addition of LC/MS grade water to reach 50% total methanol content. Solid phase extraction of samples was performed on Waters Oasis PRiME HLB 30 mg/1 cc solid phase extraction (SPE) cartridge. The cartridges were pre-conditioned with 1 mL of 50% methanol prior to loading. Samples were washed with 1 mL of 5% methanol, eluted with 500 μL acetonitrile:methanol (90:10) and dried down under a vacuum. Samples were reconstituted in 100 μL of 1% formic acid in acetonitrile and then separated by reversed-phase liquid chromatography using a Thermo Scientific™ UltiMate™ 3000 RSLC system linked to Thermo Scientific Q Exactive mass spectrometer. A Kinetex 2.6 μm XB-C18 100 Å, 100 × 1.0 mm column was used isocratically with 90% methanol in acetonitrile containing 5 mM ammonium acetate and 0.1% of acetic as the mobile phase at 50 µL/min. Full-scan product ion spectra of analytes were acquired using parallel reaction monitoring at 17,500 resolution, using 5^e5^ automatic gain control (AGC) target, 50 ms ion time and 1.3 Th isolation window. Normalized collision energies were optimized for each individual species*.*

### Oxylipins

Plasma (250 μL) was mixed with 5 μL of butylated hydroxytoluene in methanol (10 mg/mL) and 5 µL of 1 μg/mL deuterated oxylipin IS mix. Proteins were precipitated by addition of 750 μL of ice-cold methanol with 2% formic acid and chilled on ice for 30 min. Samples were centrifuged at 10,800 rpm for 10 min at 4 °C. The supernatant of each sample was collected, transferred into a 1.5 mL low retention microcentrifuge tube and then the supernatant was loaded onto a Waters Oasis PRiME HLB 30 mg/1 cc SPE cartridge. Solid phase extraction cartridges were pre-conditioned with 1 mL methanol containing 2% formic acid, 1 mL water with 2% formic acid and finally 1 mL 75% methanol consisting of 2% formic acid. Flow-through was collected in a 15 mL conical centrifuge tube and supplemented with 500 μL water and 2% formic acid. Subsequently, samples were reloaded onto the cartridges and the flow-through was further diluted by the addition of 1.5 mL water + 2% formic acid. After loading the diluted samples, the cartridges were washed with 500 μL of 5% methanol, 2% formic acid, dried down and samples were eluted with 500 μL of 90% acetonitrile, 10% methanol, 2% formic acid in microcentrifuge tubes containing 16 μL of 30% glycerol. Samples were dried under a gentle stream of nitrogen and reconstituted in 100 μL of 2% formic acid in 40% acetonitrile. Samples were filtered through 0.2 μm centrifugal filters (Thermo Scientific) and transferred to autosampler vials. Oxylipins were separated by reversed-phase liquid chromatography on a Thermo Scientific™ UltiMate™ 3000 RSLC system combined to a Thermo Scientific Q Exactive mass spectrometer using a Kinetex 2.6 μm XB-C18 100 Å, 100 × 1.0 mm column, where solvent A contained 5% ACN and solvent B consisted of 95% ACN with an addition of 0.1% of acetic acid as modifier in both mobile phases. Oxylipins were separated performing gradient elution at 100 µL/min, where mobile phase composition started out at 45% B, then by 11 min the percentage of solvent B gradually increased to 55% and finally the column was re-equilibrated for 4 min at 45% B. Full-scan product ion spectra of analytes were acquired using parallel reaction monitoring at 17,500 resolution, AGC target was set to 5^e5^ with maximum IT time of 200 ms and isolation window of 1.3 m/z. Normalized collision energies were optimized for each individual species.

### Lipid analysis and quantification

Using a method previously described [23], peak areas corresponding to lipids of interest were detected and integrated using the Tracefinder™ software (Thermo Scientific) to quantify their concentrations. The relative quantification of each lipid from each class was calculated using the IS-based normalization strategy with the known IS concentration spiked in each sample. We detected several major classes of phospholipids: phosphatidylcholine (PC), phosphatidylethanolamine (PE), lyso-PC (LPC); neutral lipids included triglycerides (TG), diglycerides (DG), cholesterol esters (CE). We also examined sphingomyelin (SM), ceramides (Cer) and hexosylceramides (HexCer) from the sphingolipid class. For each sample, the coefficient of variance (CV) from triplicates was calculated for each lipid and lipids with an average of CV > 25% were excluded from further analysis with exception of lipids containing AA and/or DHA. Regarding the known role of AA and DHA in inflammation [24, 25], lipids were categorized based on their content in AA or DHA by adding up values for either all AA-containing lipids or all those containing DHA. Given the link between lipid unsaturation and chronic cardiovascular and other health conditions with an inflammatory component [26, 27] lipids were subdivided based on their number of double bonds; saturated fatty acids (SFA) were calculated by adding lipids with no double bonds, monounsaturated fatty acids (MUFA) were calculated by adding lipids with one double bond, and polyunsaturated fatty acids (PUFA) were calculated by adding lipid species with 3 or more double bonds. Analyses of major group comparisons were restricted to lipid species with at least 64% coverage within the dataset.

### Statistics

Group differences on general demographics were evaluated with Fisher exact test to accommodate groups with < 5 counts. Normality of each lipid class categorization group and each individual species was determined by plotting histograms, as well as by numerically assessing the skewness and kurtosis values. Non-normally distributed data were log transformed for parametric analyses. Lipidomic data were analyzed using mixed linear modeling (MLM) as described previously [23, 28] to examine the independent effects of sex, ME/CFS diagnosis, and any potential interaction between them. These analyses help account for random noise associated with repeated measurements and are found suitable for lipidomics and metabolomics datasets [29–31]. Sidak post hoc analyses were performed to correct the false discovery rate (FDR) due to multiple testing. Hierarchical clustering was performed for individual lipids with a coverage superior to 85%, using the webtool MetaboAnalyst 5.0. After log-transformation of our dataset, clusters were created based on the degree of similarity of lipids as determined by Euclidean distance. Lipids that were close in distance based on the Ward algorithm were combined in clusters and an analysis of variance (ANOVA) was applied to select the top 50 lipids significantly modulated by sex and diagnosis which were then represented as dendrogram and heatmap. Principal component analysis was performed for the top 50 lipids obtained from Metaboanalyst and used severity measures of classic symptoms experienced by those with ME/CFS, including combined joint and muscle pain (physical pain), headache, cognitive difficulties and fatigue. Severity ratings for each of these reported symptoms was based on participant responses to the DePaul Fatigue questionnaire, in which a combined report of joint and muscle pain quantified physical pain severity, and the rest of the symptom severities was determined from direct ratings provided for headaches, cognitive difficulties, and fatigue. Correlation analyses were also performed on ethanolamides and oxylipins with severity of above-mentioned symptoms. For all PCA and correlation analyses, adjusted *p*-value < 0.05 after Benjamini and Hochberg (B-H) correction was considered significant.

## Results

### Sex stratified analyses show significant differences between controls and ME/CFS patients for major lipid classes

Our cohort included cases and controls of similar age and body mass index (BMI) with 40 females and 38 males among Whites, 1 female and 6 males among Asians and 9 females and 6 males among African Americans (both Asians and African Americans are referred as non-Caucasians). Given the known influence of sex on ME/CFS diagnosis, all analyses were stratified by male and female sexes (see Table 1 for general demographics).

**Fig. 1:**
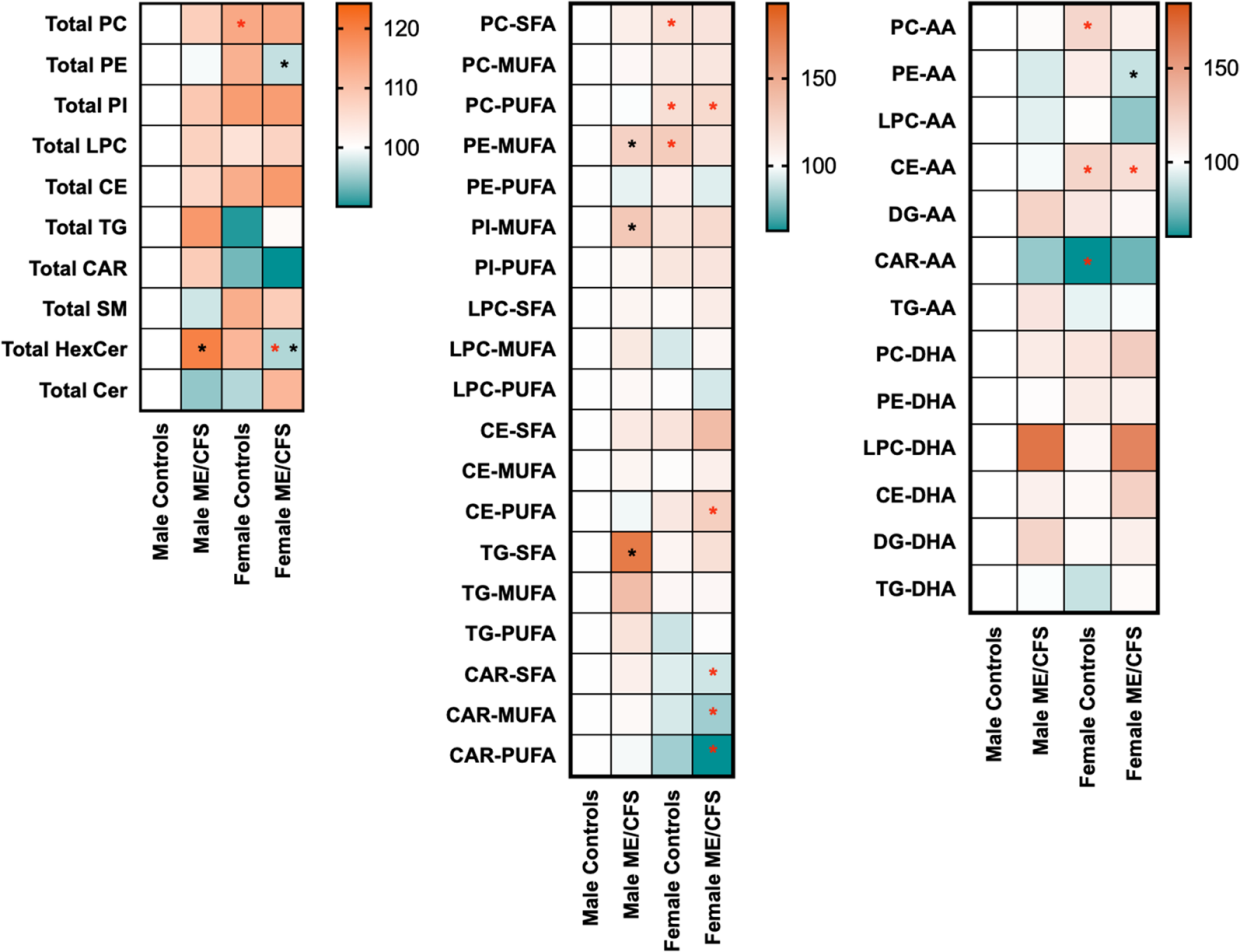
Heatmap visualization of phospholipids and neutral lipids in plasma of ME/CFS patients and controls. The heatmaps represent the average of relative concentrations to male controls of the total level of each lipid classes (left), lipids stratified by their level of unsaturation (middle) and lipids containing arachidonic acid (AA) or docosahexaenoic acid (DHA) (right) in each group. Black asterisks show significant differences between male and female ME/CFS patients and controls (p < 0.05) and red asterisks show significant differences between males and females within the same diagnosis status (p < 0.05)

Figure 1 shows a heatmap of global lipid profiles that are differentially affected by sex and diagnostic differences among males and females. Relative to healthy males, a significant increase in the total PC content was detected in healthy females (*p* < 0.05). Sex stratified analyses showed that among males, there was a significant increase in total HexCer levels in ME/CFS patients compared to controls (*p* < 0.05). Among females, ME/CFS patients had lower levels of PE and HexCer when compared to controls (*p* < 0.05). Given the association between lipid unsaturation status and inflammation in chronic health conditions, we examined the degree of unsaturation of PL and NL and showed no differences in female ME/CFS compared to female controls. However, among males, PE-MUFA, PI-MUFA and TG-SFA, were significantly higher in ME/CFS patients compared to controls (*p* < 0.05). Due to the respective pro-inflammatory and anti-inflammatory properties of AA and DHA metabolites, profiles of these fatty acids in each applicable lipid class were examined. These results showed that AA- and DHA-containing PC and CE were elevated whereas CAR-AA was decreased in healthy females compared to healthy males (*p* < 0.01). Among females, PE-AA were decreased in ME/CFS patients compared to controls (*p* < 0.01). Levels of CE-AA were elevated in female ME/CFS compared to male ME/CFS patients.

Because of the numerical increase in DHA consumption in females compared to males and the well-known effect of DHA supplementation on lipid levels, analyses were also performed by excluding individuals with known DHA consumption. While most associations described above remained unaltered even after this adjustment, additional differences were detected where total CAR was significantly decreased and total Cer was significantly elevated in female ME/CFS compared to male ME/CFS patients. The decrease of total PE in female ME/CFS compared to female controls was no longer statistically significant after adjusting for DHA consumption (Additional file 1: Figure S1). For the degree of unsaturation of lipids, while most differences remained significant, differences between male and female ME/CFS for PE-MUFA, CAR-SFA, and CAR-MUFA were no longer statistically significant as well as those observed between male ME/CFS and male controls for TG-SFA. The exclusion of individuals taking DHA revealed a significant decrease of CAR-PUFA in female controls compared to male controls (Additional file 2: Figure S2). Significant increases in PC-DHA were detected among female ME/CFS compared to male ME/CFS (Additional file 3: Figure S3) which were attributed to an elevation of PC 40:6, PC 40:7 and PC 38:7 in female ME/CFS (Additional file 5: Figure S5). Only the PC-AA, PC 40:4, showed an increase in female controls compared to male controls (*p* = 0.032) and a decrease in female ME/CFS patients compared to female controls (*p* = 0.005, Additional file 4: Figure S4). Unlike PC-DHA, the decrease of PE-AA in female ME/CFS compared to female controls was influenced by DHA consumption (Additional file 4: Figure S4).

### Bioactive lipid metabolites are differentially modulated by sex and ME/CFS diagnosis

**Fig. 2:**
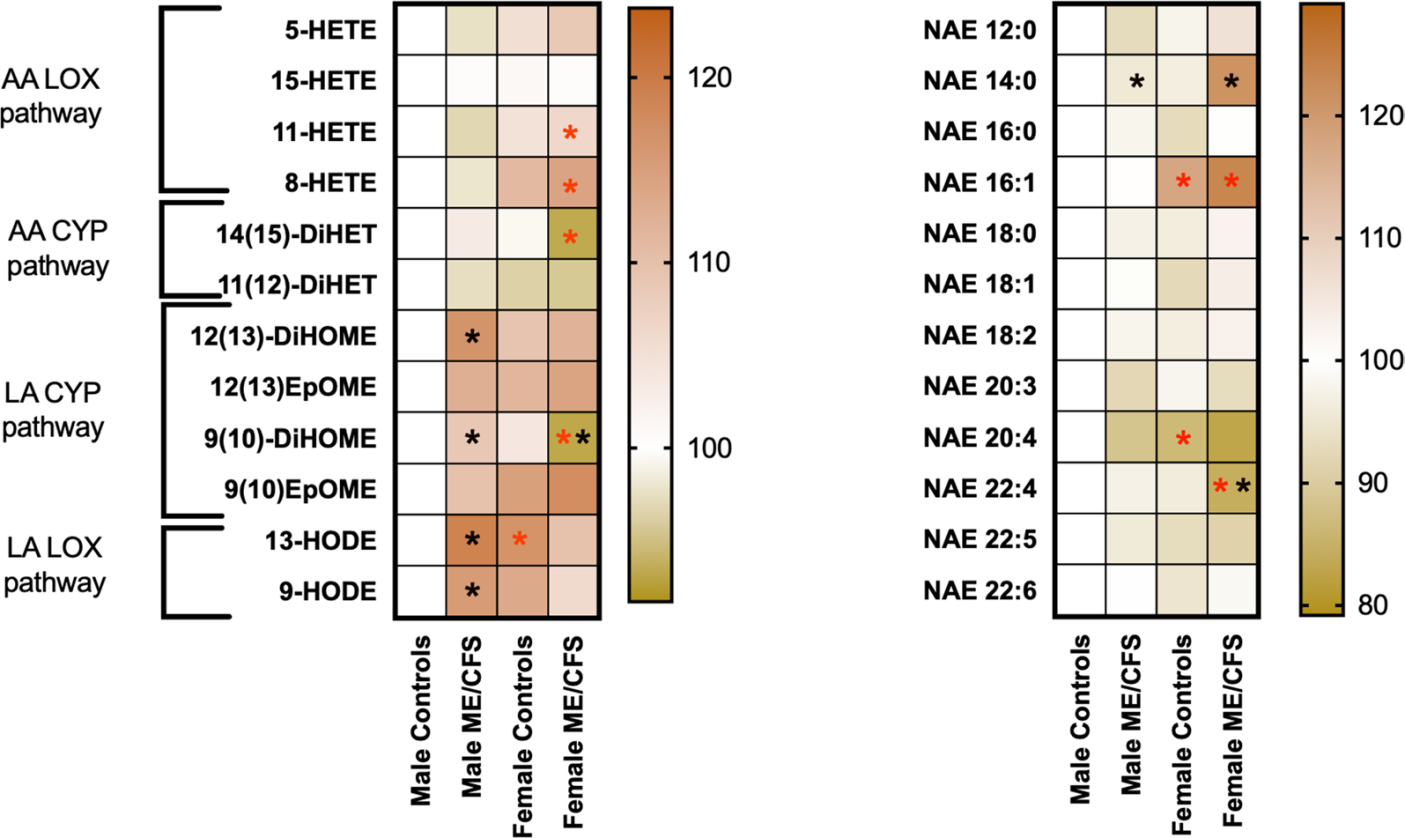
Heatmap visualization of bioactive lipids in plasma of ME/CFS patients and controls. The heatmaps represent the average of relative concentrations to male controls of oxylipins (left) and ethanolamides (right) in each group. Oxylipins are classified based on which fatty acid they are derived, arachidonic acid (AA) or linoleic acid (LA) and which pathway they derived from, lipoxygenase pathway (LOX pathway) or cytochrome P450 pathway (CYP pathway). Black asterisks show significant differences between male and female ME/CFS patients and controls (p < 0.05) and red asterisks show significant differences between males and females within the same diagnosis status (p < 0.05)

Given the implication of immunity and inflammation in ME/CFS, we investigated plasma oxylipin and ethanolamide levels because of their well-known role in these biological processes. We observed that AA-derived oxylipins were influenced by sex but did not show significant differences between ME/CFS patients and their respective controls (Fig. 2). Among males, linoleic acid derived oxylipins 12(13)-DiHOME, 9(10)-DiHOME, 13-HODE and 9-HODE were elevated in ME/CFS patients compared to controls. While 13-HODE levels were increased between female and male controls, 9(10)-DiHOME was the only LA-derived oxylipin to be decreased in female ME/CFS *versus* female controls. Similarly, the AA-containing ethanolamide, NAE 20:4, was decreased in female controls compared to male controls. Compared to their respective controls, NAE 14:0 was lower in male ME/CFS but elevated in female ME/CFS patients. Level of NAE 22:4 was lower both in female ME/CFS compared to female controls as well as in female ME/CFS compared to male ME/CFS. After excluding those with DHA supplementation, elevation of 8-HETE in female ME/CFS compared to male ME/CFS and 9(10)-DiHOME elevation in male ME/CFS compared to controls were no longer significant. Except for 9(10)-DiHOME, remaining comparisons between male and female ME/CFS and their respective controls remained unaffected (Additional file 6: Figure S6). As with NAE, while NAE 14:0 was no longer significant, NAE 18:0 was significantly elevated in female ME/CFS compared to female controls (Additional file 6: Figure S6). Additional oxylipins related to AA metabolism that were not included in the main analysis due to low coverage are provided as Additional file 7: Figure S7.

### Hierarchical clustering analysis identify sex-specific lipid species signature of ME/CFS

**Fig. 3:**
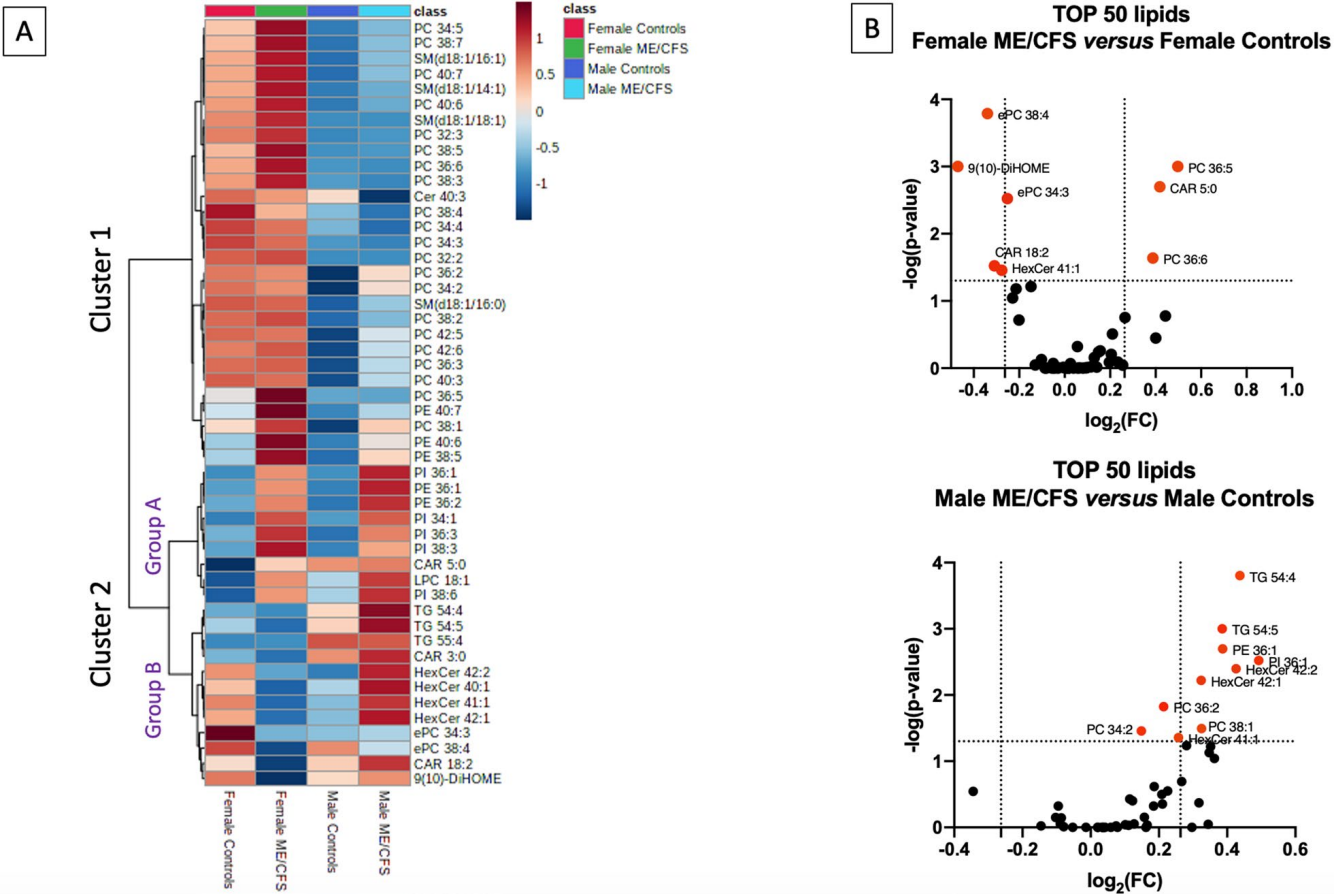
Hierarchical clustering and relative levels the of TOP 50 lipids. The scaled concentration value of each lipid from the TOP 50 identified by MetaboAnalyst are projected on level heatmap for each group. The color legend identifies in red the lipids in high level and blue the lipids at lower concentration (**A**). Relative changes in females (top) and males (bottom) between ME/CFS patients and their respective controls are represented on the Volcano plot (**B**). Each dot represents one individual lipid from the TOP 50 identified by the clustering and the red dots indicate the significant changes relative to controls with *p*-value threshold set at 0.05 (horizontal dotted line). The vertical dotted lines represent the arbitrary fold-change (FC) cut-off of ± 1.2

Given the observed influence of DHA on blood lipid composition, clustering analyses were performed after exclusion of DHA taking individuals. These analyses identified 50 lipids organized in two clusters, where cluster 1 largely contained PC, PE and SM species and cluster 2 contained a mixture of CAR, PL TG, and HexCer (Fig. 3a). Lipids within cluster 1 were mostly affected by sex differences, irrespective of the ME/CFS diagnosis. However, cluster 2 had two distinct subsets with one subgroup (group A) having lipids grouped by diagnostic differences irrespective of sex and the second (group B) where lipid changes were influenced by sex and diagnosis. Further evaluation of species within each cluster showed that most of the lipids in cluster 1 were significantly increased in females compared to males (see Fig. 3A). Lipid species in cluster 2 group A were higher in ME/CFS patients irrespective of sex while lipids of group B were decreased in females. All lipids from clusters 1 and 2 that showed a significant difference between ME/CFS patients and controls with similar sex proportion were represented in Fig. 3B. When we looked at the significance level of lipids from cluster 1, only PC 36:5 and PC 36:6 were significantly increased in female ME/CFS *versus* female controls while PC 36:2 was increased in male ME/CFS compared to male controls (Fig. 3B). In group A from cluster 2, a significant increase of CAR 5:0 was observed in ME/CFS compared to controls among females. Among males, PI 36:1 and PE 36:1 species were increased in ME/CFS patients compared to controls. The majority of lipids within group B were significantly decreased in female ME/CFS patients compared to male ME/CFS patients, especially HexCers and TGs. Among females, ePC 38:4, ePC 34:3, CAR 18:2, and 9(10)-diHOME and HexCer 41:1 species were decreased in ME/CFS patients compared to controls (Fig. 3B). In males, TG and HexCer levels were significantly higher in ME/CFS patients versus controls. Only HexCer 41:1 species showed changes both between male ME/CFS patients and male controls and female ME/CFS patients and female ME/CFS (−log(*p-value*) = 1.357 for males and −log(*p-value*) = 1.456 for females) but in opposite directions.

### Lipid species associated with ME/CFS symptoms

Principal component analysis was performed to examine an association between lipids and ME/CFS symptoms. This analysis identified 3 major components that explained 55.55% of the total variance in the top 50 plasma lipids described above (Table 2).

**Table 2:** Principal components from the principal component analysis of the TOP 50 lipids

Component	Variance	% of variance	Cumulative variance %	Headache (*p-*value)	Physical pain (*p-*value)	Cognitive difficulties (*p-*value)	Fatigue (*p-*value)
1	18.757	37.515	37.515	0.111	0.273	0.144	0.284
2	4.714	9.428	46.943	**0.026**	0.835	**0.04**	**< 0.001**
3	4.306	8.611	55.555	**0.001**	**0.027**	**0.028**	**0.004**

The three components that explain half of the variance of the lipid data are listed in the table. The association of each component with headache, physical pain, cognitive difficulties or fatigue was evaluated using fixed effect. Significant relation between component and symptoms are indicated by underlined p-value in bold, after B–H correction

As a complement to the PCA, regression analyses of the component 1 (PC1), component 2 (PC2) and component 3 (PC3) were performed to determine the effect of headaches, physical pain, cognitive difficulties and fatigue on lipid levels associated with these components. Significant influences of headache (p = 0.013), cognitive difficulties (*p* = 0.030) and fatigue (*p* < 0.001) were observed with PC2 while lipids from PC3 were modulated by physical pain (*p* = 0.027), headache (*p* < 0.001), cognitive difficulties (*p* = 0.021) and fatigue (*p* = 0.002) (Table 2). Correlations between ME/CFS symptoms and lipids contained in each 3 components are shown in Table 3.

**Table 3:** Spearman correlation of lipids identified by PCA with ME/CFS symptoms

PC1					PC2					PC3				
Lipids	Headache	Physical pain	Cognitive difficulties	Fatigue	Lipids	Headache	Physical pain	Cognitive difficulties	Fatigue	Lipids	Headache	Physical pain	Cognitive difficulties	Fatigue
PC 32:2	− 0.039	0.178	− 0.073	0.217	PC 40:6	− 0.127	0.090	− 0.244	− 0.464**	ePC 34:3	− 0.409*	− 0.295	0.09	− 0.097
PC 32:3	− 0.065	0.185	− 0.071	0.085	PC 38:7	− 0.315	− 0.082	− 0.271	− 0.471**	PC 36:2	− 0.338	− 0.124	0.018	0.021
PC 34:4	− 0.079	0.172	− 0.166	0.024	PC 40:7	− 0.271	− 0.152	− 0.271	− 0.471**	ePC 38:4	− 0.254	− 0.108	0.133	0.008
PC 34:5	− 0.105	0.120	− 0.180	− 0.188	PC 36:5	− 0.184	0.012	− 0.254	− 0.393*	PC 34:2	− 0.244	− 0.021	0.118	0.206
PC 34:3	− 0.196	0.140	− 0.147	0.036	PC 36:6	− 0.143	0.117	− 0.273	− 0.336	PC 40:3	− 0.373	− 0.250	− 0.068	− 0.012
SM(d18:1/14:1)	− 0.260	− 0.049	− 0.301	− 0.169	PC 38:5	− 0.179	− 0.04	− 0.316	− 0.398*	PC 36:3	− 0.226	0.010	0.052	0.158
PC 36:6	− 0.143	0.117	− 0.273	− 0.336	PC 42:6	− 0.122	− 0.161	− 0.237	− 0.252	SM(d18:1/16:0)	− 0.356	− 0.118	− 0.098	− 0.264
PC 36:3	− 0.226	0.010	0.052	0.158	SM(d18:1/16:0)	− 0.356	− 0.118	− 0.098	− 0.264	PC 38:2	− 0.201	− 0.069	− 0.139	− 0.007
PC 38:2	− 0.201	− 0.069	− 0.139	− 0.007	PC 34:5	− 0.105	0.120	− 0.180	− 0.188	PC 38:1	− 0.192	− 0.163	− 0.044	− 0.094
PC 34:2	− 0.244	− 0.021	0.118	0.206	SM(d18:1/16:1)	− 0.311	− 0.115	− 0.209	− 0.265	SM(d18:1/16:1)	− 0.311	− 0.115	− 0.209	− 0.265
PI 36:3	− 0.179	− 0.032	− 0.122	0.039	PC 38:1	− 0.192	− 0.163	− 0.044	− 0.094	PC 38:4	− 0.206	0.073	− 0.232	− 0.240
PI 34:1	− 0.102	0.085	0.017	0.269	SM(d18:1/18:1)	− 0.216	0.068	− 0.189	− 0.292	PC 34:3	− 0.196	0.140	− 0.147	0.036
PI 38:3	− 0.143	0.200	− 0.158	0.125	SM(d18:1/14:1	− 0.260	− 0.049	− 0.301	− 0.169	PC 38:3	0.008	0.252	− 0.030	0.158
SM(d18:1/16:1)	− 0.311	− 0.115	− 0.209	− 0.265	PC 42:5	− 0.093	− 0.028	− 0.210	− 0.069					
PC 36:2	− 0.338	− 0.124	0.018	0.021	PC 40:3	− 0.373	− 0.250	− 0.068	− 0.012					
Cer 40:3	− 0.048	0.144	− 0.207	0.052	LPC 18:1	− 0.352	− 0.273	− 0.081	− 0.104					
PC 38:3	0.008	0.252	− 0.030	0.158										

The coefficient of correlation of each individual lipid from the 3 principal components (PC1, PC2 and PC3) with the severity of headache, physical pain, cognitive difficulties or fatigue is reported in the table. Asterisks show significant correlations using adjusted p-values after B–H correction (*p < 0.05; **p < 0.01)

We also evaluated severity of these symptoms with the top 50 lipids identified by cluster analyses and found that many of those symptoms, specifically headache severity, cognitive difficulties, and fatigue severity, were negatively correlated with blood PL levels (Additional file 8: figure S8). Correlations between oxylipins, ethanolamides and symptom severity are presented in Additional file 9: figure S9, where most of these lipids show lower concentrations with an increase in each symptom severity.

## Discussion

This study analyzed plasma lipid profiles of ME/CFS patients and healthy controls by measuring the levels of lipid species from PL and NL classes as well as bioactive lipid metabolites, such as oxylipins and ethanolamides. Because the risk of developing ME/CFS can be up to four times higher in females [Centers for Disease Control and Prevention (CDC), 2018] and sex differences are known to influence blood lipid levels [32], lipid profiles of male and female ME/CFS patients were compared to controls. We found that, in males, total HexCer levels as well as several LA-derived oxylipins were significantly increased in ME/CFS patients compared to controls. Among females, significant decreases of PE and HexCer in ME/CFS patients compared to controls. We also show differential alterations in the degree of unsaturation of lipids and of AA and DHA in male ME/CFS and female ME/CFS patients compared to their respective controls. Since these lipids can influence inflammation and metabolic processes, a potential role of these lipids in these biological aspects are discussed below.

In eukaryotes, PE represents one of the most abundant PL accounting for up to 25% of cellular lipids [33]. It is now well-known that PE plays an important role not just in supporting cellular membranes, but also in vesicle formation and fusion [33]. There are many redundant synthesis pathways and intracellular locations where PE are synthesized, underscoring an importance of PE in biological systems. Phosphatidylethanolamines are reservoirs for DHA (omega-3) and AA (omega-6), which, on demand, can be converted to bioactive lipid metabolites that modulate inflammatory pathways [24, 25, 34]. Our results suggest a generalized decrease of PE in female ME/CFS but the degree of unsaturation of PE was unaffected by ME/CFS. Instead, AA-containing PE species were specifically lower among female ME/CFS patients, suggesting a selective loss of AA-containing PE species (which can be further metabolized to modulate inflammatory responses) in female ME/CFS patients.

Through a series of enzymatic steps, PE is converted to *N*-acylethanolamine and subsequently to ethanolamides that have anti-inflammatory properties [35]. Since ethanolamides target cannabinoid receptors that modulate pain and cognition [36], it is possible that blood NAE levels may be indicative of CNS symptoms experienced by ME/CFS patients. This is supported by our findings that among female ME/CFS patients, lower levels of several NAE species were associated with an increasing severity of CNS-related symptoms and fatigue severity. These data suggest further evaluation of the relationship between ethanolamides and ME/CFS symptoms.

Following PL hydrolysis, released AA can be metabolized to produce oxylipins that promote inflammation whereas DHA metabolites support anti-inflammatory processes [24, 25]. In our study decreases in AA-containing PE species in female ME/CFS patients suggest potential impact on AA synthesis or metabolism. Furthermore, differential alterations of AA- and DHA-containing PI, PE and PC species suggests their involvement in inflammation commonly observed in ME/CFS patients. As several plasma AA- and DHA-containing PC and PE species were negatively associated with CNS symptom severity, this may also suggest a differential regulation of these lipids with ME/CFS symptomatology. As such, additional mechanistic studies are required to better understand an association between AA and DHA containing lipids and ME/CFS.

Since metabolic products of AA include oxylipins, we also examined these lipids in relation to ME/CFS. For instance, prostaglandins (PG) have been shown to activate receptors that initiate signaling pathways involved in [37] peripheral inflammation [38]. As such, increases in their levels in ME/CFS patients may be indicative of ongoing inflammation associated with this condition. In addition to prostaglandins, AA can also be metabolized by lipoxygenase (LOX) and cytochrome P-450 (CYP) epoxygenase and ω-hydroxylase to generate AA-derived oxylipins. However, other AA-derived oxylipins did not differ between ME/CFS and controls. Instead, many LA-derived oxylipins showed significant changes, with 12(13)-DiHOME, 9(10)-DiHOME, 13-HODE and 9-HODE increased in male ME/CFS patients versus controls and 9(10)-DiHOME decreased in female ME/CFS patients compared to controls. As LA is the precursor of AA, it is a possibility that the use of LA for the synthesis of LA-derived oxylipins reduces the LA amount available for the synthesis of AA leading to a decrease of AA-derived oxylipins. A negative correlation observed between oxylipins and cognitive difficulties and physical pain among females with ME/CFS may suggest a protective role of these oxylipins in ME/CFS. Interestingly, LA-derived oxylipins have been associated with increased inflammatory pain [39] and correlate with increased levels of pro-inflammatory interleukin-6 (IL-6) in human plasma after prolonged and intensive exercise known to lead to high levels of inflammatory mediators [40]. They also reduce the number of regulatory T-cells which correlates with increased susceptibility of autoimmune and chronic inflammatory diseases [41–44]. However, as these oxylipins did not differ that much in female patients and controls, these biological changes may be relevant to male ME/CFS patients. Altogether, the changes observed in these bioactive lipids reflect ongoing inflammation associated with ME/CFS which is consistent with the literature that shows a modulation of AA and DHA containing lipids.

Sphingolipids contribute to the integrity of cell membranes and alter inflammatory and metabolic processes [45]. Ceramides, that are both metabolites and precursors of SM, are also well known for their role in modulating innate immune responses, particularly by macrophages and neutrophils [46]. Hexosylceramides are derived from Cer [47] and suppress the production of pro-inflammatory cytokines, such as tumor necrosis factor alpha (TNF-α), interleukin-1ß (IL-1ß) and IL-6, after exposure of macrophages induced by bacterial toxin [18]. While SM, as a class, was not significantly affected in our study, several SM species were identified to be different between male and female participants, irrespective of their diagnosis. Similarly, Cer did not reach significance in our current study, despite a generalized increase of about 18% in female ME/CFS patients compared to female controls. This contrasts with the study by Germain and colleagues [48, 49] which showed a significant increase in several Cer species in female ME/CFS patients. Instead, we observed that Hexcer were increased in ME/CFS patients, irrespective of their sex. Prior studies have shown that HexCer levels are elevated in patients with chronic hepatitis C infection and autoimmune diseases [48, 49]. While a role of these sphingolipids remains to be studied in ME/CFS, the above referenced studies do suggest that sphingolipid changes may be associated with autoimmune- and infection-related immune and inflammatory changes observed in ME/CFS patients. Therefore, an association between sphingolipid metabolites in ME/CFS warrants further investigation.

Among male ME/CFS patients, PE-MUFA, PI-MUFA and TG-SFA appeared to be increased. While the biological relevance of these findings remains to be elucidated, in general, the presence of saturated lipids can contribute to inflammation associated with chronic diseases, such cardiovascular disease [50]. As such, elevated TG-SFA in male ME/CFS patients may require further investigation. Many aspects of metabolic disorders lie with bioenergetic deficiencies [51]. Since fatty acids represent an energy rich source for cellular bioenergetics, their transport into mitochondria for beta-oxidation requires prior activation by carnitine, converting them into CAR which can then enter mitochondria [52]. As such, CAR levels can be seen as indicators of fatty acid oxidation and deficiencies in their blood levels can reflect an altered metabolic state in the body. While there were no overall diagnostic differences in CAR levels, hierarchical clustering analyses point to possible differences in CAR 5:0 and CAR 18:2 in ME/CFS patients. Significance of these specific lipids remains unknown and additional studies are needed. Hence, our data do not suggest fatty acid transport as a contributor to mitochondria dysfunction that is often attributed to ME/CFS [53]. However, other specific markers of mitochondrial lipid metabolism, such as cardiolipins, were not evaluated in this study owing to insufficient and low levels of these lipids in plasma as they are largely present in the intracellular mitochondria.

## Limitations

A limitation of the study is that a few ME/CFS subjects were on DHA supplements, for which we adjusted our analyses. It is now known that diets supplemented with omega-3, including DHA, have an effect on lipids, in particular, high density lipoprotein cholesterol (HDL-C), non-HDL-C and TGs [54–56]. In this study, DHA supplementation did not change the directions of lipid changes in relation to sex and ME/CFS diagnosis but affected the statistical significance of sex-related lipid differences, with a minimal effect on lipid variations associated with ME/CFS diagnosis. Changes could also have been driven by additional clinical characteristics or medications taken by subjects for which we had no record. These confounds prevented us from determining the potential influence of outliers within the study. Additionally, as plasma lipids may represent mixed contributions of several tissues and organs, the association between plasma lipid changes and CNS symptoms requires further attention to better understand CNS involvement in ME/CFS symptom presentation and etiology.

## Conclusion

In summary, by designing a study integrating sex as a biological variable, we have been able to account for its effects to detect sex-specific ME/CFS lipid profiles. We also identified sex-specific differences in plasma lipids irrespective of diagnostic status which may help better explain the role of sex on lipid processing in future studies. Many lipids evaluated here may be implicated in inflammation and immunity as well as metabolic responses. Since lipid metabolism in blood immune cells appears critical for their function, evaluation of lipid profiles in immune cells will help better understanding their role in the ongoing disease processes of ME/CFS. As we observed an association of plasma lipids with severity of fatigue, headache and cognitive difficulties, additional studies are also needed to evaluate the role of these lipids in the CNS component of ME/CFS. Further studies in larger cohorts are required to confirm the significance of these lipid variations in ME/CFS pathogenesis, particularly in relation to immune dysfunction.

## Supplementary Information

**Additional file 1: Figure S1.** Histograms of total level of phospholipids (PL) and neutral lipids (NL) after exclusion of DHA taking individuals. The graphs represent the average concentrations of PL and NL relative to male controls. Black asterisks show significant differences between male and female ME/CFS patients and controls (p < 0.05) and red asterisks show significant differences between males and females within the same diagnosis status (p < 0.05).

**Additional file 2: Figure S2.** Histograms of level of lipids by level of unsaturation after exclusion of DHA taking individuals. The graphs represent the average concentrations of saturated (SFA), monounsaturated (MUFA) and polyunsaturated (PUFA) lipids relative to male controls. Black asterisks show significant differences between male and female ME/CFS patients and controls (p < 0.05) and red asterisks show significant differences between males and females within the same diagnosis status (p < 0.05).

**Additional file 3: Figure S3.** Histograms of total level of AA/DHA-containing lipids after exclusion of DHA taking individuals. The graphs represent the average concentrations of lipids relative to male controls. Black asterisks show significant differences between male and female ME/CFS patients and controls (p < 0.05) and red asterisks show significant differences between males and females within the same diagnosis status (p < 0.05).

**Additional file 4: Figure S4.** Histograms of individual AA-containing lipids after exclusion of DHA taking individuals. The graphs represent the average concentrations of AA-containing lipids relative to male controls. Black asterisks show significant differences between male and female ME/CFS patients and controls (p < 0.05) and red asterisks show significant differences between males and females within the same diagnosis status (p < 0.05).

**Additional file 5: Figure S5.** Histograms of level of individual DHA-containing lipids after exclusion of DHA taking individuals. The graphs represent the average concentrations of DHA-containing lipids relative to male controls. Black asterisks show significant differences between male and female ME/CFS patients and controls (p < 0.05) and red asterisks show significant differences between males and females within the same diagnosis status (p < 0.05).

**Additional file 6: Figure S6.** Histograms of bioactive lipids after exclusion of DHA taking individuals. The graphs represent the average concentrations of oxylipins (top) and ethanolamides (bottom) relative to male controls. Black asterisks show significant differences between male and female ME/CFS patients and controls (p < 0.05) and red asterisks show significant differences between males and females within the same diagnosis status (p < 0.05).

**Additional file 7: Figure S7.** Heatmap visualization of oxylipins with low coverage in plasma of ME/CFS patients and controls. The heatmaps represent the average of relative concentrations to male controls of AA-derived oxylipins with a coverage comprised between 5 and 52% in each group.

**Additional file 8: Figure S8.** Spearman correlation of TOP 50 lipids with ME/CFS symptoms. The coefficient of correlation of each lipid with the severity of headache, physical pain, cognitive difficulties or fatigue is reported on the heatmap. The color legend identifies in red the lipids that negatively correlate with each symptom and in blue the lipids that have a positive correlation. Pink asterisks show significant correlations (p < 0.05).

**Additional file 9: Figure S9.** Spearman correlation of bioactive lipids in females (left) and males (right). The coefficient of correlation of ethanolamides and oxylipins with the severity of headache, physical pain, cognitive difficulties or fatigue is reported on the heatmap. The color legend identifies in red the lipids that negatively correlate with each symptom and in blue the lipids that have a positive correlation. Pink asterisks show significant correlations (p < 0.05).

## Data Availability

Not applicable.

## Abbreviations

AA: Arachidonic acid
AGC: Automatic gain control
ANOVA: Analysis of variance
BMI: Body mass index
CAR: Acylcarnitines
CE: Cholesterol ester
Cer: Ceramides
CNS: Central nervous system
COX: Cyclooxygenase
CSF: Cerebrospinal fluid
CV: Coefficient of variance
CYP: Cytochrome P-450
DG: Diglycerides
DHA: Docosahexaenoic acid
DSM-IV criteria: Diagnostic and Statistical Manual of Mental Disorders criteria
HDL-C: High-density lipoprotein cholesterol
HexCer: Hexosylceramides
HPLC/MS: High-performance liquid chromatography-mass spectrometry
ICH-GCP: International Committee on Harmonization of Good Clinical Practice
IL-1ß: Interleukin-1ß
IL-6: Interleukin-6
IRB: Institutional Review Board
IS: Internal standards
LA: Linoleic acid
*LC/MS*: Liquid chromatography mass spectrometry
LOX: Lipoxygenase
LPC: Lysophosphatidylcholines
ME/CFS: Myalgic encephalomyelitis/chronic fatigue syndrome
MUFA: Monounsaturated
NAE: *N*-Acylethanolamine
NK: Natural killer
NL: Neutral lipids
nLC: Nano-flow liquid chromatography
Non-HDL-C: Non-high-density lipoprotein cholesterol
PC: Phosphatidylcholine
PCA: Principal component analysis
PE: Phosphatidylethanolamine
PEM: Post-exertional malaise
PG: Prostaglandins
PI: Phosphatidylinositol
PL: Phospholipids
PUFA: Polyunsaturated fatty acids
QC: Quality control
SFA: Saturated fatty acid
SM: Sphingomyelin
SPE: Solid phase extraction
TG: Triglycerides
TNF-ɑ: Tumor necrosis factor alpha
Tregs: T-regulatory
