# Supplementary figures and images for: Sex-specific plasma lipid profiles of ME/CFS patients and their association with pain, fatigue, and cognitive symptoms
Source: J Transl Med. 2021 Aug 28;19:370. doi: 10.1186/s12967-021-03035-6 (PMC8401202; doi:10.1186/s12967-021-03035-6)

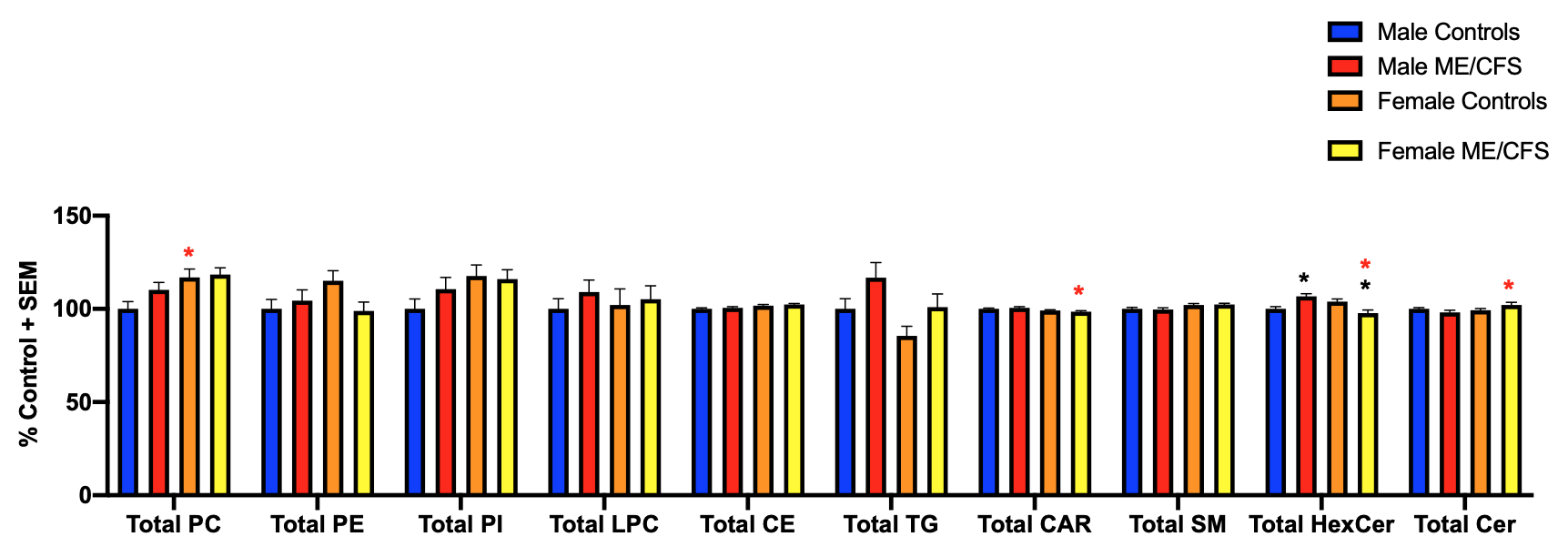

Supplement: Supplementary file 1 — Additional file 1: Figure S1. Histograms of total level of phospholipids (PL) and neutral lipids (NL) after exclusion of DHA taking individuals. The graphs represent the average concentrations of PL and NL relative to male controls. Black asterisks show significant differences between male and female ME/CFS patients and controls (p < 0.05) and red asterisks show significant differences between males and females within the same diagnosis status (p < 0.05). [file 12967_2021_3035_MOESM1_ESM.tiff]

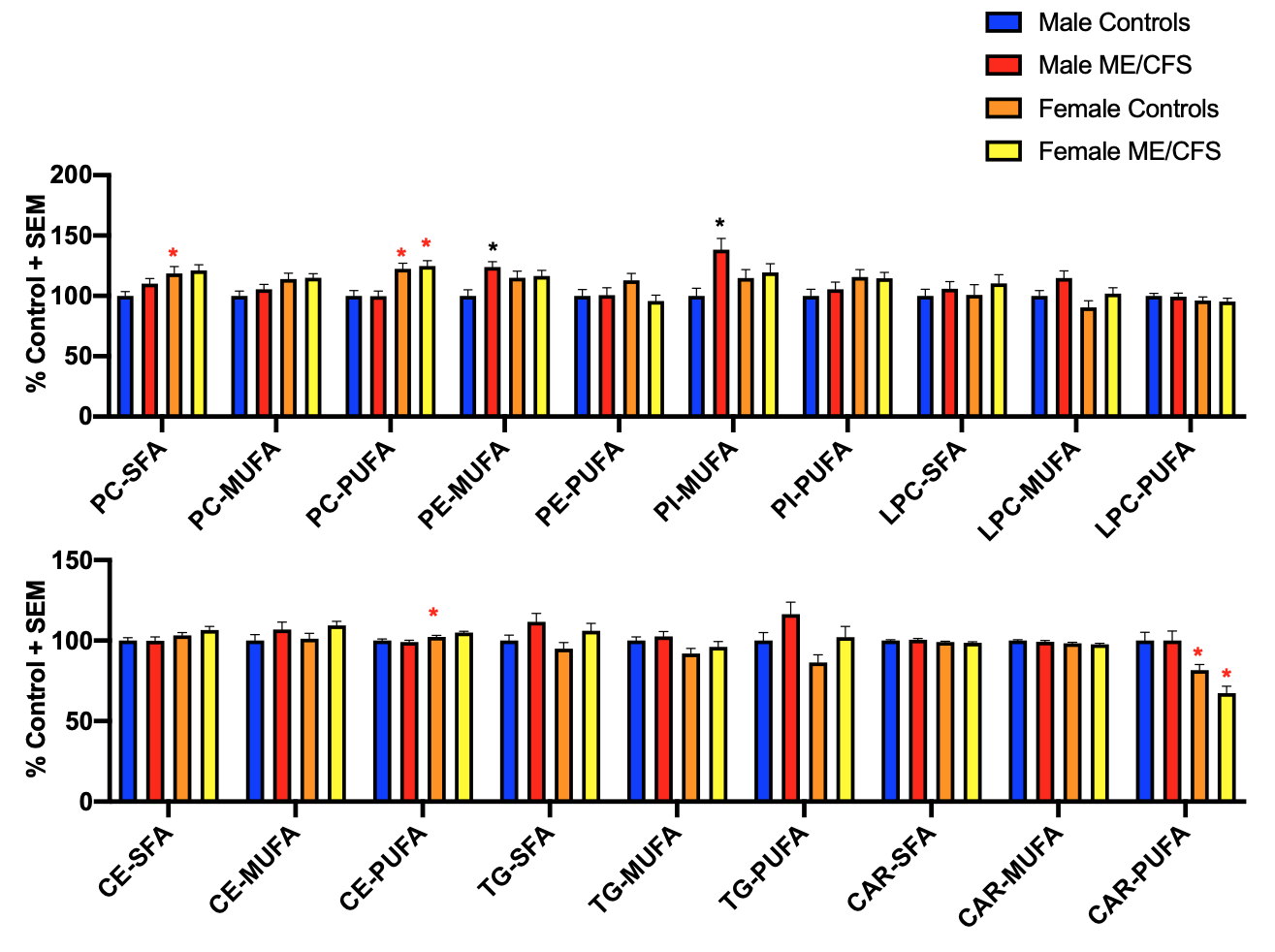

Supplement: Supplementary file 2 — Additional file 2: Figure S2. Histograms of level of lipids by level of unsaturation after exclusion of DHA taking individuals. The graphs represent the average concentrations of saturated (SFA), monounsaturated (MUFA) and polyunsaturated (PUFA) lipids relative to male controls. Black asterisks show significant differences between male and female ME/CFS patients and controls (p < 0.05) and red asterisks show significant differences between males and females within the same diagnosis status (p < 0.05). [file 12967_2021_3035_MOESM2_ESM.tiff]

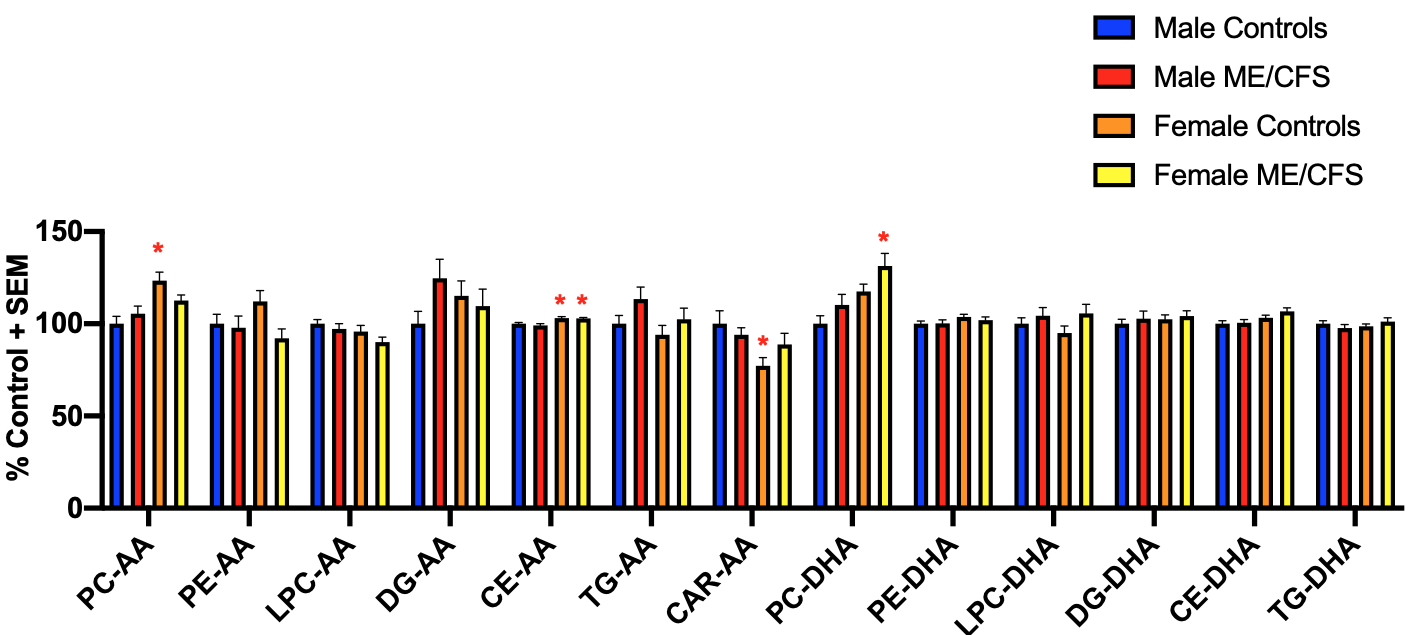

Supplement: Supplementary file 3 — Additional file 3: Figure S3. Histograms of total level of AA/DHA-containing lipids after exclusion of DHA taking individuals. The graphs represent the average concentrations of lipids relative to male controls. Black asterisks show significant differences between male and female ME/CFS patients and controls (p < 0.05) and red asterisks show significant differences between males and females within the same diagnosis status (p < 0.05). [file 12967_2021_3035_MOESM3_ESM.tiff]

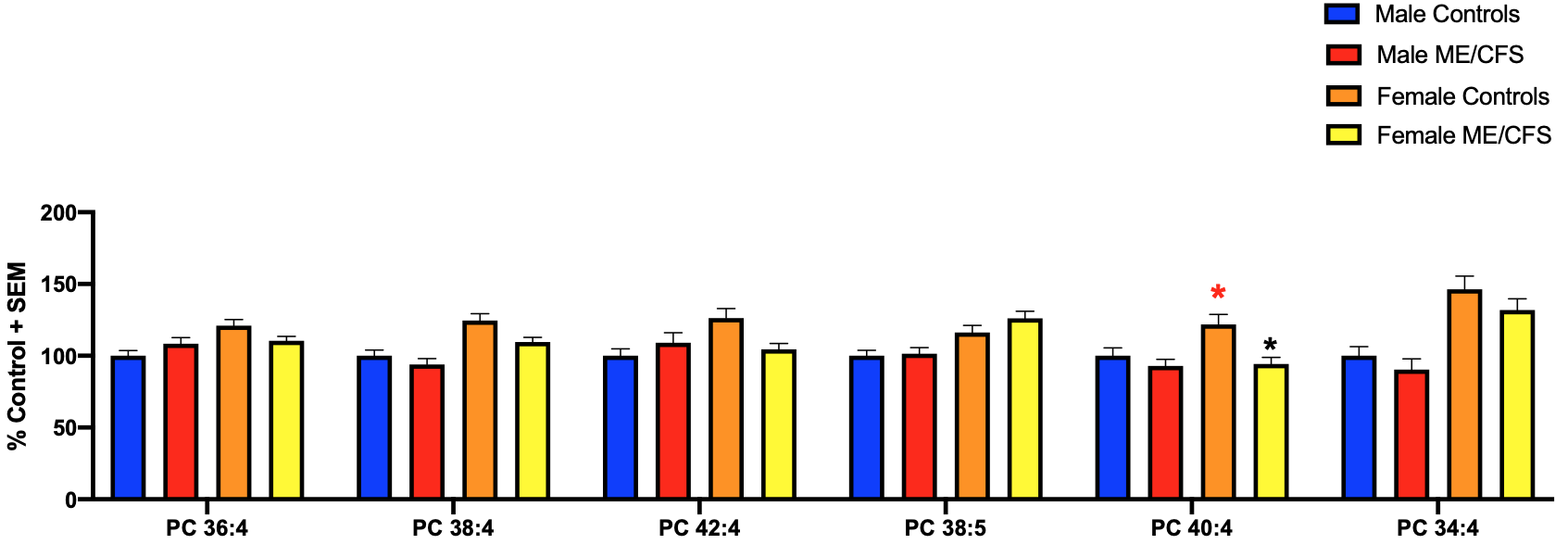

Supplement: Supplementary file 4 — Additional file 4: Figure S4. Histograms of individual AA-containing lipids after exclusion of DHA taking individuals. The graphs represent the average concentrations of AA-containing lipids relative to male controls. Black asterisks show significant differences between male and female ME/CFS patients and controls (p < 0.05) and red asterisks show significant differences between males and females within the same diagnosis status (p < 0.05). [file 12967_2021_3035_MOESM4_ESM.tiff]

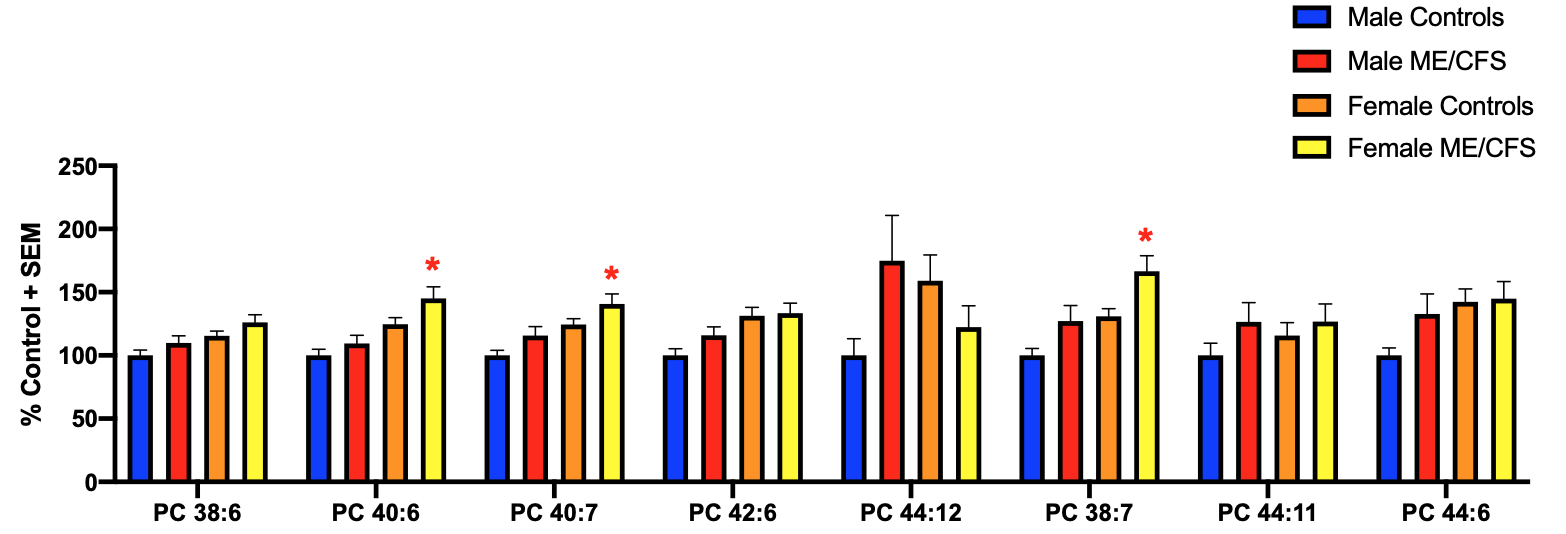

Supplement: Supplementary file 5 — Additional file 5: Figure S5. Histograms of level of individual DHA-containing lipids after exclusion of DHA taking individuals. The graphs represent the average concentrations of DHA-containing lipids relative to male controls. Black asterisks show significant differences between male and female ME/CFS patients and controls (p < 0.05) and red asterisks show significant differences between males and females within the same diagnosis status (p < 0.05). [file 12967_2021_3035_MOESM5_ESM.tiff]

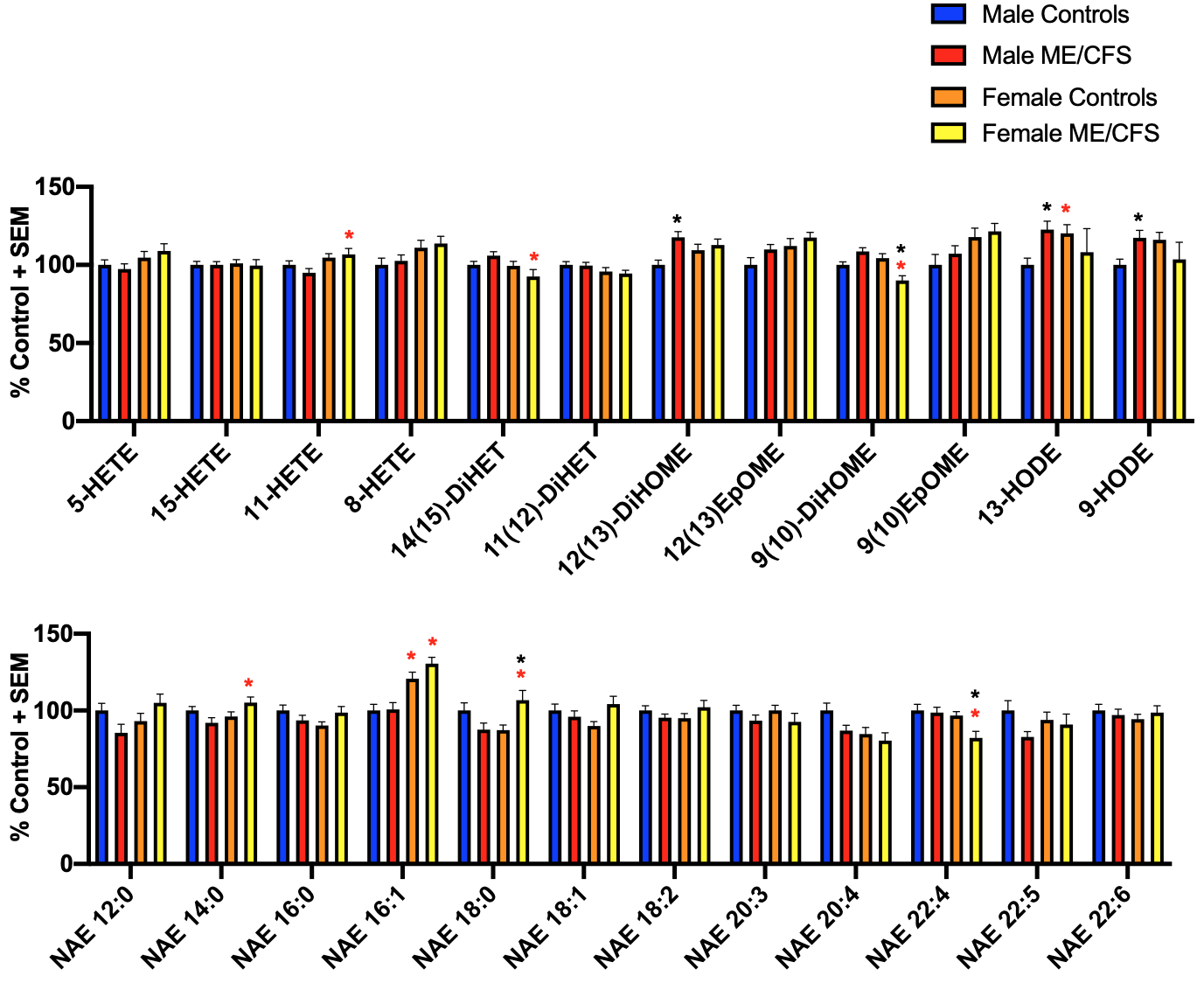

Supplement: Supplementary file 6 — Additional file 6: Figure S6. Histograms of bioactive lipids after exclusion of DHA taking individuals. The graphs represent the average concentrations of oxylipins (top) and ethanolamides (bottom) relative to male controls. Black asterisks show significant differences between male and female ME/CFS patients and controls (p < 0.05) and red asterisks show significant differences between males and females within the same diagnosis status (p < 0.05). [file 12967_2021_3035_MOESM6_ESM.tiff]

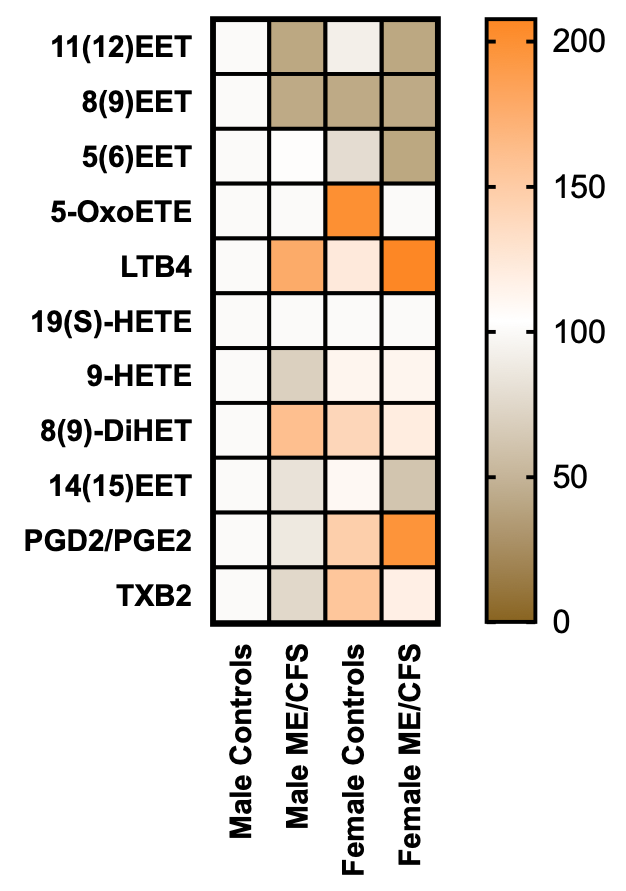

Supplement: Supplementary file 7 — Additional file 7: Figure S7. Heatmap visualization of oxylipins with low coverage in plasma of ME/CFS patients and controls. The heatmaps represent the average of relative concentrations to male controls of AA-derived oxylipins with a coverage comprised between 5 and 52% in each group. [file 12967_2021_3035_MOESM7_ESM.tiff]

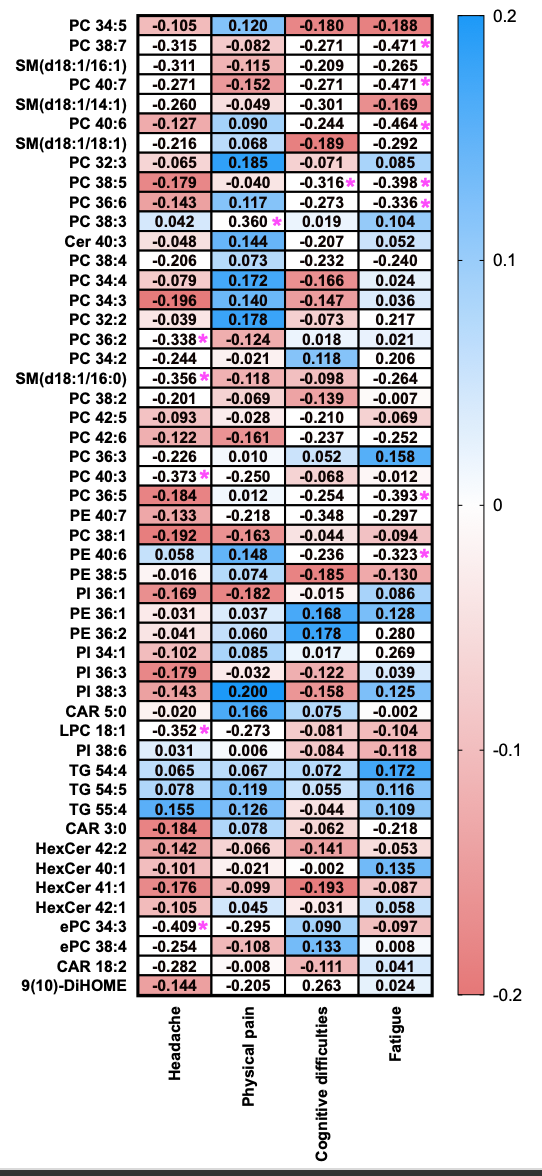

Supplement: Supplementary file 8 — Additional file 8: Figure S8. Spearman correlation of TOP 50 lipids with ME/CFS symptoms. The coefficient of correlation of each lipid with the severity of headache, physical pain, cognitive difficulties or fatigue is reported on the heatmap. The color legend identifies in red the lipids that negatively correlate with each symptom and in blue the lipids that have a positive correlation. Pink asterisks show significant correlations (p < 0.05). [file 12967_2021_3035_MOESM8_ESM.tiff]

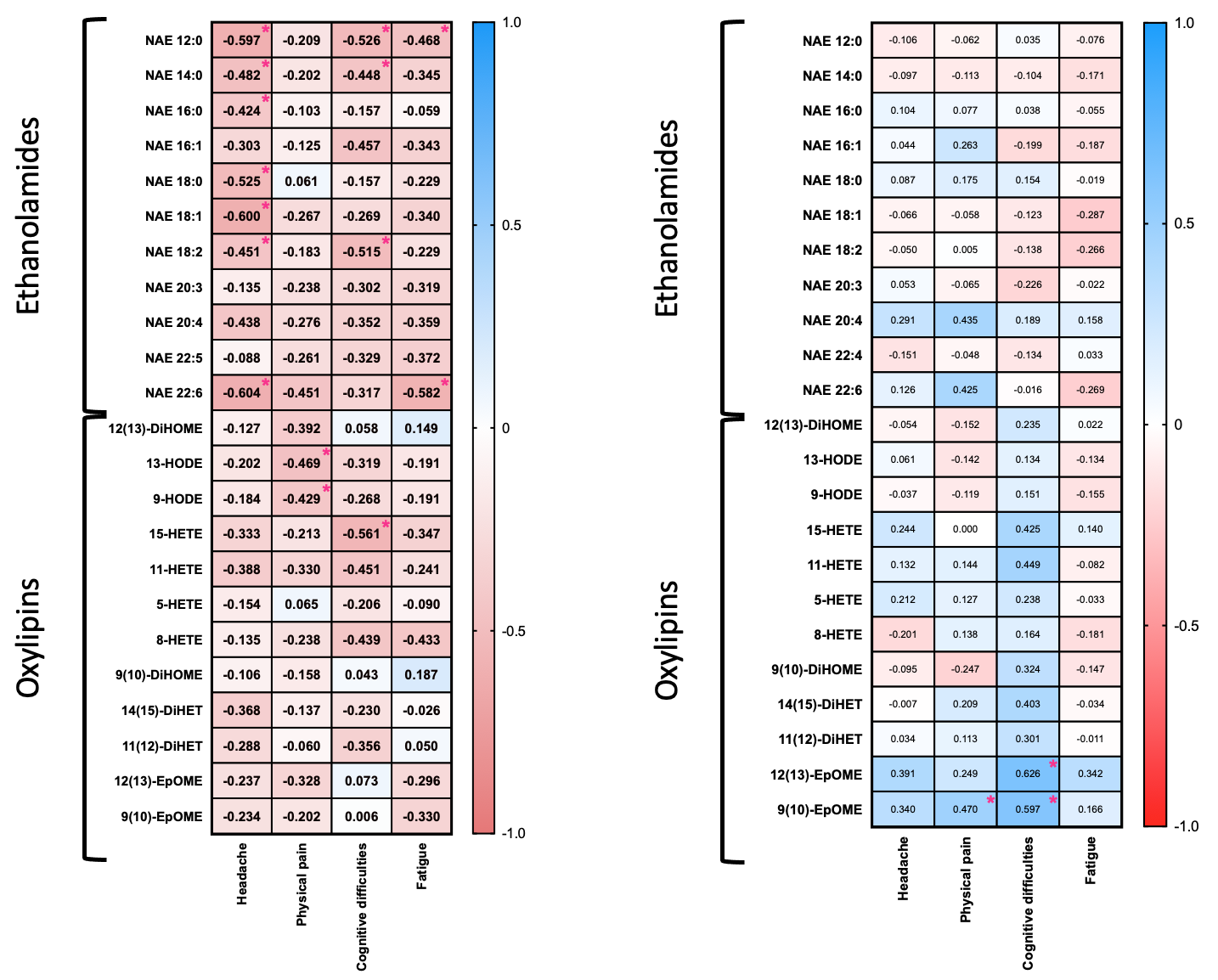

Supplement: Supplementary file 9 — Additional file 9: Figure S9. Spearman correlation of bioactive lipids in females (left) and males (right). The coefficient of correlation of ethanolamides and oxylipins with the severity of headache, physical pain, cognitive difficulties or fatigue is reported on the heatmap. The color legend identifies in red the lipids that negatively correlate with each symptom and in blue the lipids that have a positive correlation. Pink asterisks show significant correlations (p < 0.05). [file 12967_2021_3035_MOESM9_ESM.tiff]
